# P2Y_6_-deficiency increases micturition frequency and attenuates sustained contractility of the urinary bladder in mice

**DOI:** 10.1038/s41598-017-00824-2

**Published:** 2017-04-10

**Authors:** Satoru Kira, Mitsuharu Yoshiyama, Sachiko Tsuchiya, Eiji Shigetomi, Tatsuya Miyamoto, Hiroshi Nakagomi, Keisuke Shibata, Tsutomu Mochizuki, Masayuki Takeda, Schuichi Koizumi

**Affiliations:** 1 Department of Urology, University of Yamanashi Graduate School of Medical Science, Yamanashi, 409-3898 Japan; 2 Department of Neuropharmacology, University of Yamanashi Graduate School of Medical Science, Yamanashi, 409-3898 Japan; 3 Japan Science and Technology Agency, CREST, Tokyo, 102-0076 Japan

## Abstract

The role of the P2Y_6_ receptor in bladder function has recently attracted a great deal of attention in lower urinary tract research. We conducted this study to determine contributions of the P2Y_6_ receptor in lower urinary tract function of normal phenotypes by comparing P2Y_6_-deficient mice and wild-type mice. In *in vivo* experiments, P2Y_6_-deficient mice had more frequent micturition with smaller bladder capacity compared to wild-type mice; however, there was no difference between these groups in bladder-filling pressure/volume relationships during cystometry under decerebrate, unanaesthetized conditions. Analysis of *in vivo* bladder contraction revealed significant difference between the 2 groups, with P2Y_6_-deficient mice presenting markedly shorter bladder contraction duration but no difference in peak contraction pressure. However, analysis of *in vitro* experiments showed no P2Y_6_ involvements in contraction and relaxation of bladder muscle strips and in ATP release by mechanical stimulation of primary-cultured urothelial cells. These results suggest that the P2Y_6_ receptor in the central nervous system, dorsal root ganglion, or both is involved in inhibition of bladder afferent signalling or sensitivity in the pontine micturition centre and that the receptor in the detrusor may be implicated in facilitation to sustain bladder contraction force.

## Introduction

P2Y receptors are G-protein-coupled receptors for extracellular nucleotides^1^, which are classified into eight P2Y receptor subtypes (P2Y_1_, P2Y_2_, P2Y_4_, P2Y_6_, P2Y_11–14_)^2–4^. P2Y receptors are found in various animal species, implying their development early in evolution^5^.

The P2Y_6_ receptor is a uridine diphosphate (UDP)-preferring receptor and is partially responsive to uridine-5′-triphosphate (UTP) and adenosine diphosphate (ADP) but not responsive to adenosine triphosphate (ATP)^4, 6^. The receptor couples to G_q_ and induces inositol lipid signalling through phospholipase C-β isozymes^6^. The P2Y_6_ receptor is widely distributed in various tissues including placenta, thymus, spleen, kidney, vascular smooth muscle, lung, small intestine, bone, fat cells, spinal cord and brain^2, 4, 7^, and is expressed in many kinds of cells such as intestinal epithelial cells, T cells (affected T cells), monocytes, microglia, vascular endothelial cells, cardiomyocytes, smooth muscle cells and neurones^2, 4, 7–9^. Thus, this receptor is implicated in a variety of pathophysiological conditions such as immune responses, pro-inflammatory responses, astrocyte apoptosis, effector T cell activation, phagocytosis, neuropathic pain^2, 4, 10–12^. Extensive studies have suggested that the use of P2Y_6_ antagonists may be a useful strategy to treat diseases related to inflammation, neurodegeneration and nociception^10, 12–15^.

The potential role of the P2Y_6_ receptor in lower urinary tract function has been raised by recent studies that have demonstrated functional involvements of the receptor in urothelial signalling^16, 17^ and detrusor contractility^18^ of the urinary bladder. Carneiro and co-workers showed that an activation of the urothelial P2Y_6_ receptor increases voiding frequency during cystometry in the anaesthetized rat by facilitating ATP release from the bladder urothelium to stimulate suburothelial afferent nerves^16^. Yu and co-workers demonstrated that the P2Y_6_ activation by UDP enhances P2X-mediated bladder smooth muscle contractility^18^. While these studies have been conducted to determine functions of the P2Y_6_ receptor in the bladder, roles of the receptor in the central nervous system (CNS) and the peripheral nervous system (PNS) (i.e., in the reflex micturition circuitry) are still unknown. While blockade of the P2Y_6_ receptor in the bladder has been proposed to be therapeutically useful for alleviating persistent storage symptoms induced by pathological bladder^19^, the effect of the systemic receptor blockade on physiological functions needs clarification. Thus, we performed the present study using P2Y_6_-knockout (KO) mice to determine the contributions of the P2Y_6_ receptor in lower urinary tract function of wild-type (WT) phenotypes.

Recently, we have developed dual voiding function analysis of both voluntary voiding behaviour in metabolic cage and unanaesthetized reflex micturition during cystometry^20^. A combination of a genetically modified mouse and this novel approach has allowed us to perform detailed evaluation of targeted-gene phenotypes in lower urinary tract function of the small rodent. First, we evaluated *in vivo* lower urinary tract activity by dual voiding function analysis and found significant differences between P2Y_6_-KO mice and WT mice. Second, to determine whether the differences in the *in vivo* experiment results were attributable to bladder local mechanisms, we compared the 2 groups by examining the contraction and relaxation of *in vitro* bladder muscle strips in response to pharmacologic treatments and by evaluating ATP release from primary-cultured urothelial cells in response to mechanical stimulation.

## Results

### mRNA expression of P2Y_6_ and other P2 subtypes

**Figure 1:**
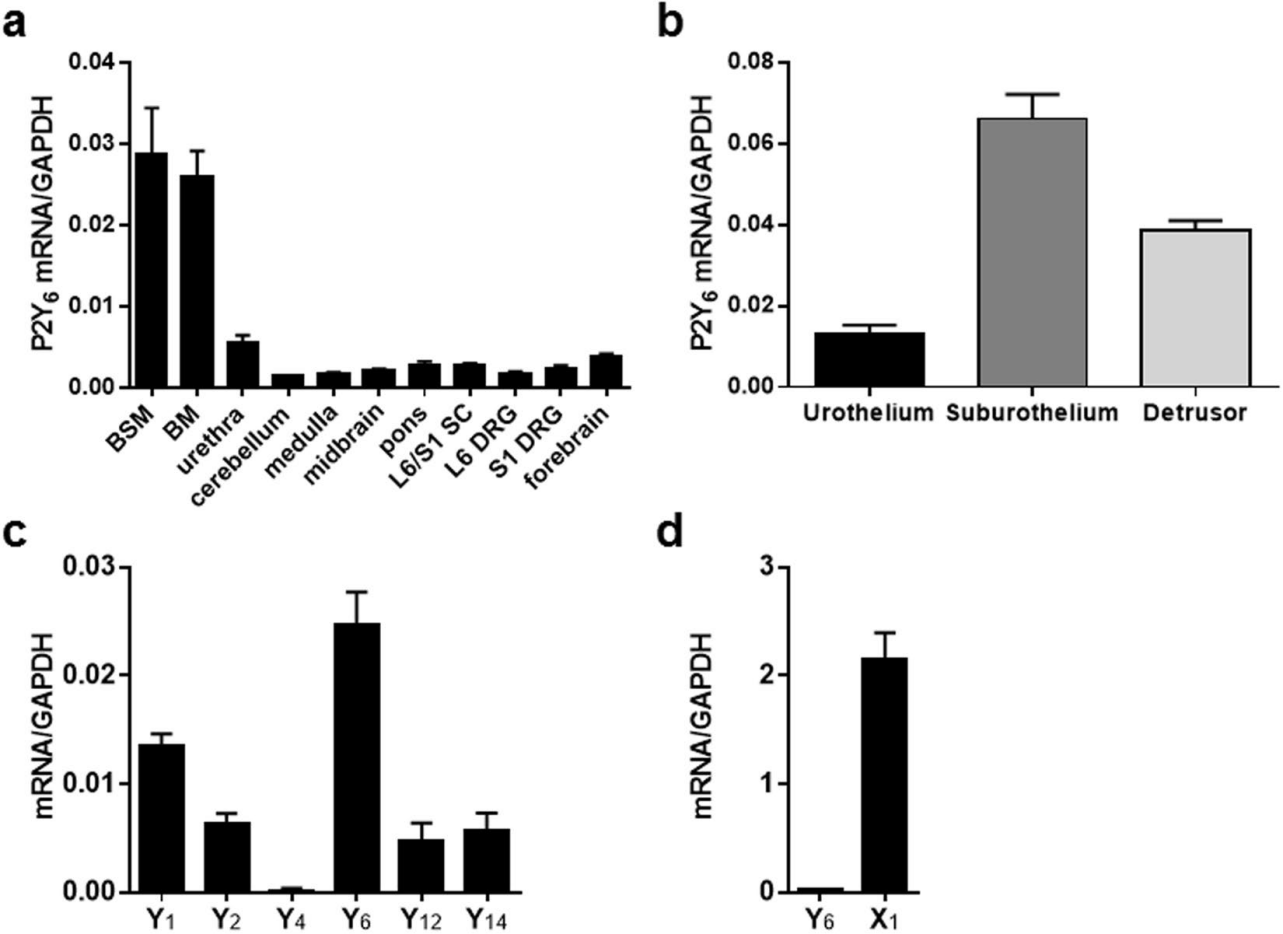
P2Y_6_ mRNA expression in the WT mouse. (**a**) Quantitative real-time RT-PCR analysis of P2Y_6_ mRNA expression in tissues associated with the control of lower urinary tract activity (n = 5). Glyceraldehyde-3-phosphate dehydrogenase (GAPDH) was used as a reference gene. BSM, bladder smooth muscle; BM, bladder mucosa; SC, spinal cord; DRG, dorsal root ganglion. (**b**) P2Y_6_ mRNA expression in urothelium, suburothelium, and detrusor (n = 7). (**c**,**d**) Gene expression of P2Y subtypes (n = 10) and P2X_1_ (n = 10). Data are presented as the mean ± SEM.

Real-time RT-PCR analysis was performed to examine P2Y_6_ mRNA expression in body tissues associated with the control of lower urinary tract function in normal (i.e., WT) mice. The expression of P2Y_6_ mRNA was widely distributed in the central nervous system (CNS), L6/S1 dorsal root ganglion (DRG), bladder and urethra (Fig. 1a). Gene expression was abundant in the lower urinary tract, especially in the bladder. The rank order of P2Y_6_ mRNA expression in the bladder was as follows: suburothelium > detrusor > urothelium (Fig. 1b). Comparisons of mRNA expression levels of P2Y_6_ and other purinergic receptor subtypes were performed in the detrusor, where we were primarily intrigued by a previous study that examined the role of P2Y_6_ in the modulation of bladder muscle tone^18^. As shown in Fig. 1c, P2Y_6_ mRNA was expressed to the greatest extent among P2Y subtypes. Furthermore, the expression level of P2X_1_ mRNA was nearly 100 times higher than that of P2Y_6_ mRNA (Fig. 1d).

Western blotting and immunostaining to determine protein expression and localization, respectively, of P2Y_6_ receptors were reserved because currently commercially available antibodies to the P2Y_6_ receptor are unreliable and lack specificity for the receptor^21^.

### Analysis of voluntary voiding behaviour in metabolic cages

**Figure 2:**
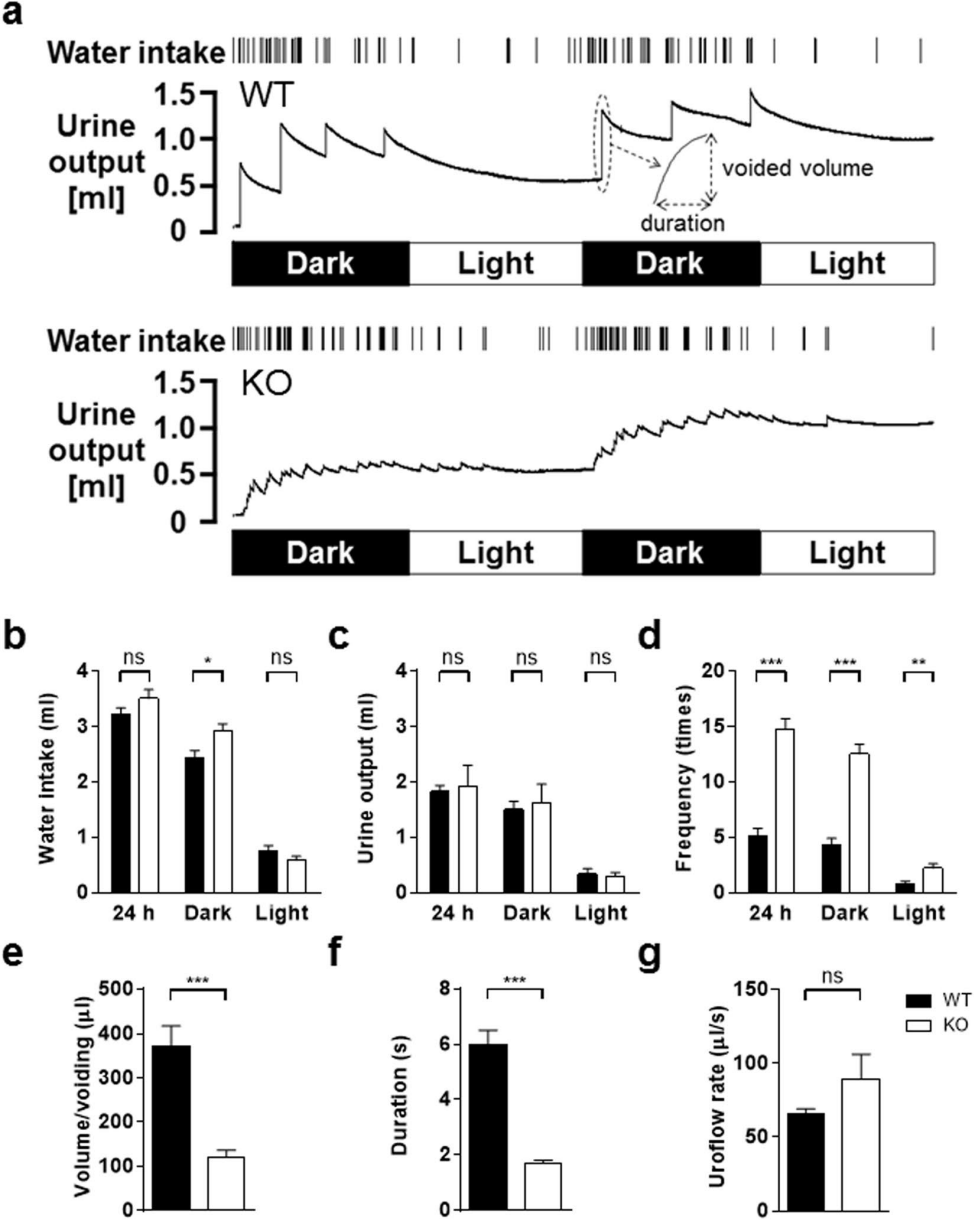
Analysis of voluntary voiding behaviour in metabolic cages. (**a**) Representative 48-h recording charts showing water intake and urine output of a WT mouse (top) and a P2Y_6_-KO mouse (bottom). Data interpretations are described in a previously published article^20^. Note that the KO mouse had a much higher voiding frequency and remarkably smaller urine volume/voiding compared with the WT mouse. Bar graphs showing quantitative analysis of metabolic cage experiments for water intake (**b**), urine output (**c**), voiding frequency (**d**), urine volume/voiding (**e**), voiding duration (**f**), and mean uroflow rate (**g**). Data are presented as the mean ± SEM. WT (n = 10) and P2Y_6_-KO (n = 10) mice were compared by unpaired t-test. *P < 0.05; **P < 0.01; ***P < 0.001; ns, not significant.

Figure 2a shows representative recording charts of 48-h voluntary voiding behaviour and water intake in a WT mouse (top) and a P2Y_6_-KO mouse (bottom). Water intake (Fig. 2b), urine output volume (Fig. 2c), and voiding frequency (Fig. 2d) were evaluated for 24 h (12 h in the dark period and 12 h in the light period). While there were no differences between the 2 groups in water intake in the light period and in 24 h, water intake in the dark period was greater in P2Y_6_-KO mice than in WT mice (Fig. 2b). No differences in urine output in any period were found between the 2 groups (Fig. 2c). In all periods, the voiding frequency of P2Y_6_-KO mice was markedly higher than that of WT mice (Fig. 2d).

Comparisons between WT and P2Y_6_-KO mice in urine volume/voiding (Fig. 2e), voiding duration (Fig. 2f), and mean uroflow rate (Fig. 2g) (i.e., voiding related variables) were performed. Urine volume/voiding in P2Y_6_-KO mice was significantly less than that in WT mice (Fig. 2e). There were no differences in urine volume/voiding between the dark period and the light period in P2Y_6_-KO mice (126.5 ± 14.5 µl and 136.1 ± 26.4 µl, respectively; P = 0.57, by a Wilcoxon matched-pairs signed rank test, n = 9) and WT mice (313.8 ± 47.8 µl and 413.4 ± 83.8 µl, respectively; P = 0.11, n = 7). Voiding duration in P2Y_6_-KO mice was significantly shorter than that in WT mice (Fig. 2f). There were no differences in mean uroflow rates between the 2 groups (P = 0.24, by Mann-Whitney test; Fig. 2g).

### Evaluation of unanaesthetized reflex activity of the lower urinary tract during cystometry

**Figure 3:**
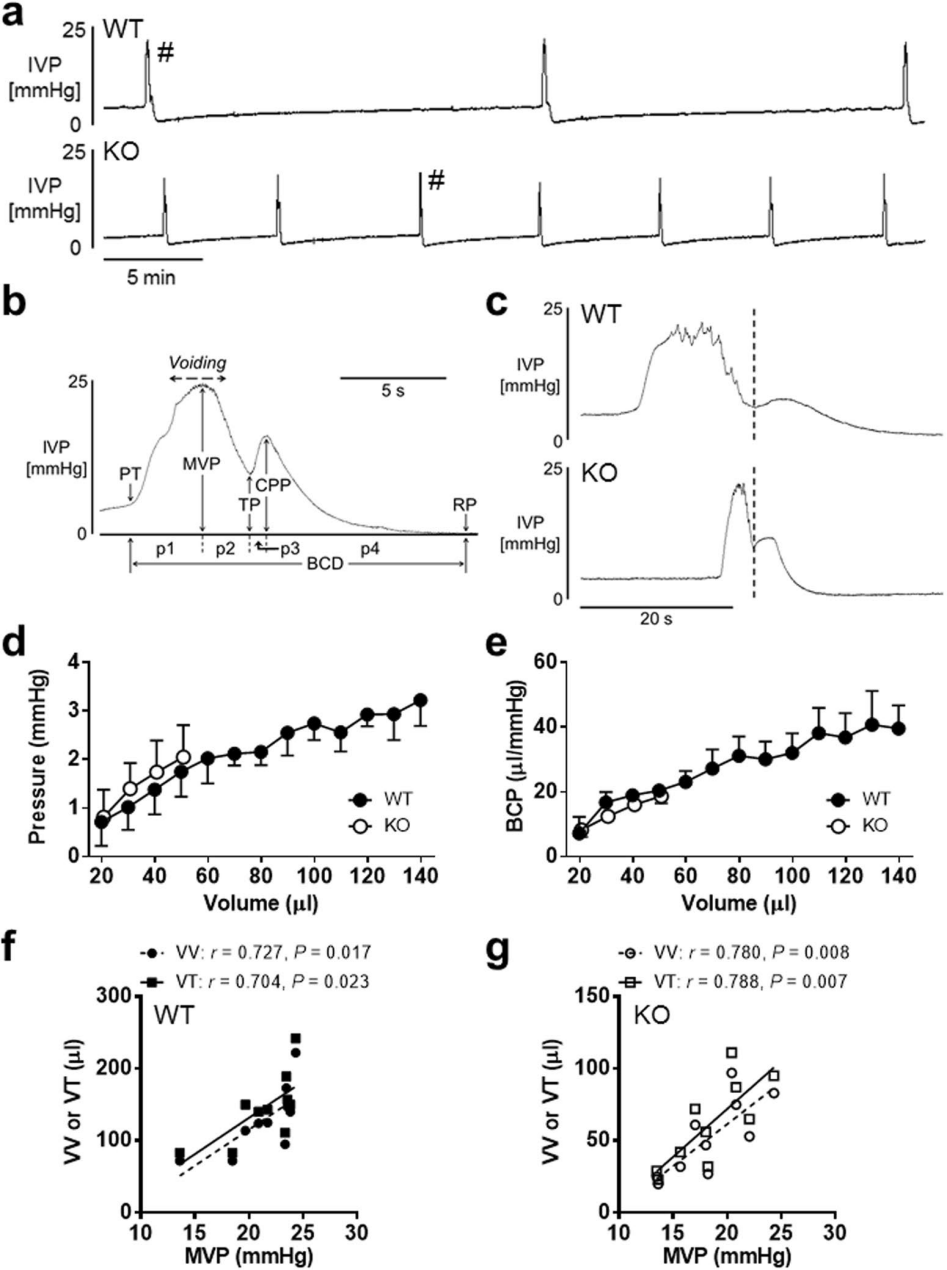
Cystometry analysis of reflex micturition cycle under decerebrate unanaesthetized conditions. (**a**) Bladder activity was recorded during continuous saline infusion cystometry (infusion rate: 10 µl/min). Note that, compared with a WT mouse (top), a P2Y_6_-KO mouse (bottom) presents increased frequency of micturition. A bladder contraction was evaluated in detail as indicated in (**b**). PT, pressure at volume threshold for micturition contraction; MVP, maximal voiding pressure; TP, trough pressure; CPP, closing peak pressure; RP, post-void resting pressure; BCD, bladder contraction duration; p1, p2, p3, and p4 are 1st, 2nd, 3rd, and 4th phases of BCD, respectively. (**c**) A comparison of a WT mouse (top) and a P2Y_6_-KO mouse (bottom), both of which were extracted from micturition contractions with the symbol (#) in (top) and (bottom), respectively, showed that a contraction duration of the P2Y_6_-KO mouse is markedly shorter than that of the WT mouse. A dotted line divides a bladder contraction into the 1st phase duration and 2nd phase duration^24^. Pressure-volume relationship (**d**) and bladder compliance (BCP)-volume relationship (**e**) of WT and P2Y_6_-KO mice showed no difference between the 2 groups (by repeated measures two-way ANOVA) and indicate a linear regression in the relationships (Y = 0.01928X + 0.6188 and Y = 0.2527X + 7.658, respectively, for WT; Y = 0.04046X + 0.08669 and Y = 0.3496X + 1.633, respectively, for P2Y_6_-KO). These relationship graphs were constructed using animals that completed values of 20 µl to 140 µl for WT and 20 µl to 50 µl for P2Y_6_-KO (n = 6 for each group). Coefficients (r) and P values (P) between MVP and VV or VT for WT (**f**) and P2Y_6_-KO (**g**) are shown (n = 10 for each group), based on the calculation by Spearman’s correlation analysis and by Pearson’s correlation analysis, respectively. VV, voided volume; VT, volume threshold for micturition contraction.

Figure 3a shows representative cystometry recordings of a WT mouse (top) and a P2Y_6_-KO mouse (bottom). Cystometry parameters evaluated in this study were described in a previously published article^20^. In addition, to provide a better evaluation of bladder contractions, we defined other parameters as shown in Fig. 3b.

P2Y_6_-KO mice were compared with WT mice in parameters associated with the bladder-filling phase and bladder contraction phase, as shown in Table 1. There were no differences between the 2 groups in increases of bladder contraction pressure such as maximal voiding pressure (MVP) and closing peak pressure (CPP; P = 0.07 and P = 0.11, respectively, unpaired t-test), whereas the bladder contraction duration (BCD) was markedly reduced in P2Y_6_-KO mice compared with WT mice (Table 1). Figure 3c shows bladder contraction traces enlarged in the abscissa of a WT mouse (top) and a P2Y_6_-KO mouse (bottom). Detailed analysis of a bladder contraction phase (Fig. 3b) showed that p1, p2 and p4 of 4 phases in BCD were markedly shorter in P2Y_6_-KO mice than those in WT mice (Table 2).

**Table 1:** Comparisons between WT and P2Y_6_-KO mice with respect to cystometric parameters during the reflex micturition cycle.

	PT (mmHg)	MVP (mmHg)	TP (mmHg)	CPP (mmHg)	RP (mmHg)	BCP (µl/mmHg)	BCD (s)	VV (µl)	RV (µl)	VT (µl)	VE (%)
WT	3.9 ± 0.3	21.3 ± 1.1	6.5 ± 0.6	8.0 ± 1.0	−0.6 ± 0.1	34.2 ± 4.7	23.3 ± 1.7	128 ± 15	16 ± 2	145±15	88 ± 2
	(2.5–5.0)	(13.6–24.3)	(3.2–8.7)	(4.1–15.1)	(−1.2–0.1)	(15.4–67.3)	(15.6–30.9)	(72–222)	(10–36)	(83–242)	(76–94)
KO	2.9 ± 0.2**	18.4 ± 1.1	8.9 ± 0.7*	10.2 ± 1.0	−0.1 ± 0.2*	19.5 ± 2.9*	14.6 ± 0.9***	52 ± 8***	9 ± 1*	61 ± 10***	84 ± 1*
	(2.0–3.6)	(13.5–24.3)	(5.5–12.4)	(4.7–13.4)	(−0.7–0.8)	(6.6–38.6)	(9.2–18.2)	(20–97)	(3–14)	(23–111)	(76–87)

PT, pressure at volume threshold for inducing micturition contraction; MVP, maximal voiding pressure; TP, trough pressure; CPP, closing peak pressure; RP, post-void resting pressure; BCP, bladder compliance; BCD, bladder contraction duration; VV, voided volume; RV, post-void residual volume; VT, volume threshold for inducing micturition contraction; VE, voiding efficiency. Numbers in parentheses are ranges. Significant difference from variable of WT mice: *P < 0.05, **P < 0.01, ***P < 0.001 (unpaired t-test or Mann-Whitney U test, according to the result of D’Agostino & Pearson omnibus normality test). Values are expressed as the mean ± SEM (n = 10 for each).

**Table 2:** Comparisons between WT and P2Y_6_-KO mice with respect to parameters associated with bladder contraction.

	p1 (s)	p2 (s)	p3 (s)	p4 (s)	p1 rise (mmHg/s)	p2 descent (mmHg/s)	p3 rise (mmHg/s)	p4 descent (mmHg/s)
WT	5.0 ± 0.8	4.2 ± 0.5	1.2 ± 0.3	12.8 ± 0.9	3.9 ± 0.4	−3.9 ± 0.5	1.8 ± 0.7	−0.7 ± 0.1
	(2.6–10.9)	(2.2–7.4)	(0.2–3.6)	(8.6–17.0)	(2.0–6.3)	(−6.6–−2.5)	(−0.5–8.0)	(−1.1–−0.4)
KO	2.4 ± 0.3**	1.8 ± 0.3***	1.4 ± 0.2	9.0 ± 0.9**	7.4 ± 1.0**	−5.4 ± 0.3*	1.0 ± 0.2	−1.2 ± 0.1***
	(1.3–3.5)	(0.5–3.5)	(0.2–2.4)	(4.9–12.6)	(3.1–12.4)	(−7.0–−3.7)	(−0.5–2.5)	(−1.5–−0.9)

The symbols of p1, p2, p3, and p4 indicate 1st, 2nd, 3rd, and 4th phases, respectively, of BCD. The p1 rise, p2 descent, p3 rise, and p4 descent are calculated as: (pressure change from PT to MVP)/p1, (pressure change from MVP to TP)/p2, (pressure change from TP to CPP)/p3, and (pressure change from CPP to RP), respectively. Numbers in parentheses are ranges. Significant difference from variable of WT mice: *P < 0.05, **P < 0.01, ***P < 0.001 (unpaired t-test or Mann-Whitney U test, according to the result of D’Agostino & Pearson omnibus normality test). Values are expressed as the mean ± SEM (n = 10 for each).

P2Y_6_-KO mice had smaller voided volume (VV) and smaller volume thresholds for inducing micturition contraction (VT) compared with WT mice (Table 1). Post-void residual volume (RV) and voiding efficiency (VE) were also smaller in P2Y_6_-KO mice than in WT mice (Table 1). The small but statistically significant reduction of VE in P2Y_6_-KO mice, despite smaller RV in P2Y_6_-KO mice compared with WT mice, was due to a greater reduction ratio in VV (59% smaller) than in VT (58% smaller).

Pressure thresholds for inducing micturition contraction (PT) and bladder compliance (BCP) in P2Y_6_-KO mice were lower than those in WT mice. However, these 2 variables were dependent on intravesical volume at the time-point of examination (Fig. 3d,e), thus suggesting there was no difference between the 2 groups in the relationship of bladder pressure or BCP against intravesical volume. Moreover, VT and VV were correlated with MVP in WT (Fig. 3f) and P2Y_6_-KO mice (Fig. 3g), showing that the bladder contraction pressure during micturition is urination volume-dependent. The results are consistent with a previous study showing a significant correlation between MVP and a variable substitute for VV or VT in male mice (but not in female mice)^22^. Small but statistically significant elevation in post-void resting pressure (RP) was detected in P2Y6-KO mice compared with WT mice; however, the underlying mechanism for the difference is unknown.

### Contractile response to carbachol, electrical field stimulation (EFS), or ATP of *in vitro* bladder muscle strips

Bladders excised from WT mice and P2Y_6_-KO mice were examined by haematoxylin and eosin (H&E) staining, which revealed that the bladder of the P2Y_6_-KO mouse presents no morphological abnormalities (e.g., thickening of muscle layer, edematous change, and granulation) in comparison with that of the WT mouse (n = 2 for each group) (Supplementary Fig. 1S). Before preparation of detrusor strips for contraction experiments, each body of bladder was weighed: the weights of WT (body weight: 25.4 ± 0.6 g) and P2Y_6_-KO (28.1 ± 0.6 g) mice were 11.6 ± 0.8 mg and 13.5 ± 1.0 mg, respectively, showing no difference between the two groups (n = 16 for each group, P = 0.14, unpaired t-test).

**Figure 4:**
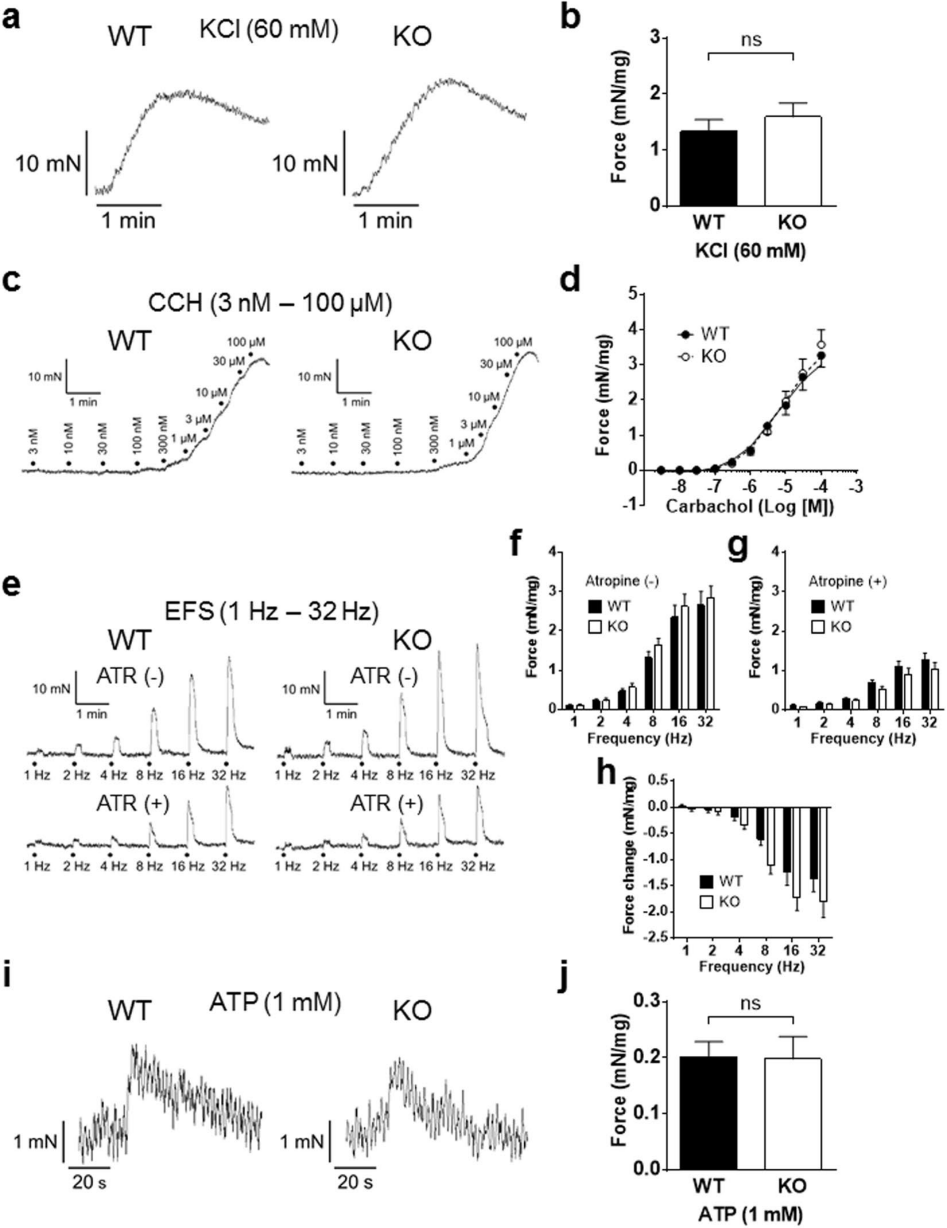
Contractile responses to KCl, carbachol, electrical field stimulation (EFS) or ATP, of *in vitro* bladder muscle strips. (**a**) A contraction of bladder muscle strip from a WT mouse (left) or a P2Y_6_-KO mouse (right) was evoked by 60 mM KCl to test its viability. No difference in contraction force evoked in response to 60 mM KCl was found between WT (n = 7) and P2Y_6_-KO (n = 9) mice (P = 0.66, Mann-Whitney U test) (**b**). (**c**) A contraction of bladder muscle strip from a WT mouse (left) or a P2Y_6_-KO mouse (right) in response to graded concentrations of carbachol (3 nM to 100 µM). Concentration-response curves of WT (n = 9) and P2Y_6_-KO (n = 7) mice were compared, showing no differences between the 2 groups (P = 1.00, Mann-Whitney U test) (**d**). (**e**) EFS-evoked contractions of bladder muscle strips from a WT mouse (left) and a P2Y_6_-KO mouse (right) (by EFS at 1 Hz, 2 Hz, 4 Hz, 8 Hz, 16 Hz, and 32 Hz) were compared in the absence or presence of atropine (ATR, 10 µM). There were no differences between the 2 groups (WT, n = 7 and P2Y_6_-KO, n = 9) in contraction forces evoked by EFS in either the absence (P = 0.79, repeated measures two-way ANOVA) (**f**) or presence (P = 0.54) (**g**) of atropine. In addition, no difference in force change by atropine of contraction evoked by EFS was found between the 2 groups (P = 0.18) (**h**). (**i**) A contraction of bladder muscle strip from a WT mouse (left) or a P2Y_6_-KO mouse (right) was evoked by 1 mM ATP. There were no differences between WT (n = 7) and P2Y_6_-KO (n = 9) mice in contraction forces evoked in response to 1 mM ATP (P = 0.73) (**j**).

Parasympathetic innervation to the bladder muscle produces, with acetylcholine and ATP, a bladder contraction during which the micturition occurs. First, an effect of 60 mM KCl was examined to demonstrate the viability of bladder muscle strips, showing no difference between WT and P2Y_6_-KO mice (Fig. 4a,b).

To compare WT and P2Y_6_-KO mice in bladder muscle strip contractility responding to cholinergic stimulation, the effect of carbachol (3 nM to 100 µM) was examined and the concentration-response curves were constructed (Fig. 4c,d). No difference was found between the 2 groups (P = 0.69, F-test).

Contractions were evoked by electrical field stimulations (at 1 Hz, 2 Hz, 4 Hz, 8 Hz, 16 Hz and 32 Hz) in the presence or absence of atropine (10 µM) to compare WT and P2Y_6_-KO mice in muscarinic contributions to the generation of the contraction (Fig. 4e). No difference in EFS-evoked contractions in either the presence or absence of atropine was found between the 2 groups (Fig. 4f,g). In addition, there was no difference between the 2 groups in the degree of contraction force changed by atropine (Fig. 4h).

Furthermore, a contraction evoked by ATP (1 mM) of the bladder muscle strip was compared between a WT mouse and a P2Y_6_-KO mouse (Fig. 4i). There were no differences between the 2 groups in peak contraction force (Fig. 4j) and in duration between the time-points of the peak contraction force and the subsequent baseline, showing 33.1 ± 6.0 s for WT mice and 35.1 ± 6.4 s for P2Y_6_-KO mice (P = 0.96, by Mann-Whitney test).

### Relaxing effect of β-adrenergic agonist on a pre-contracted *in vitro* bladder muscle strip

**Figure 5:**
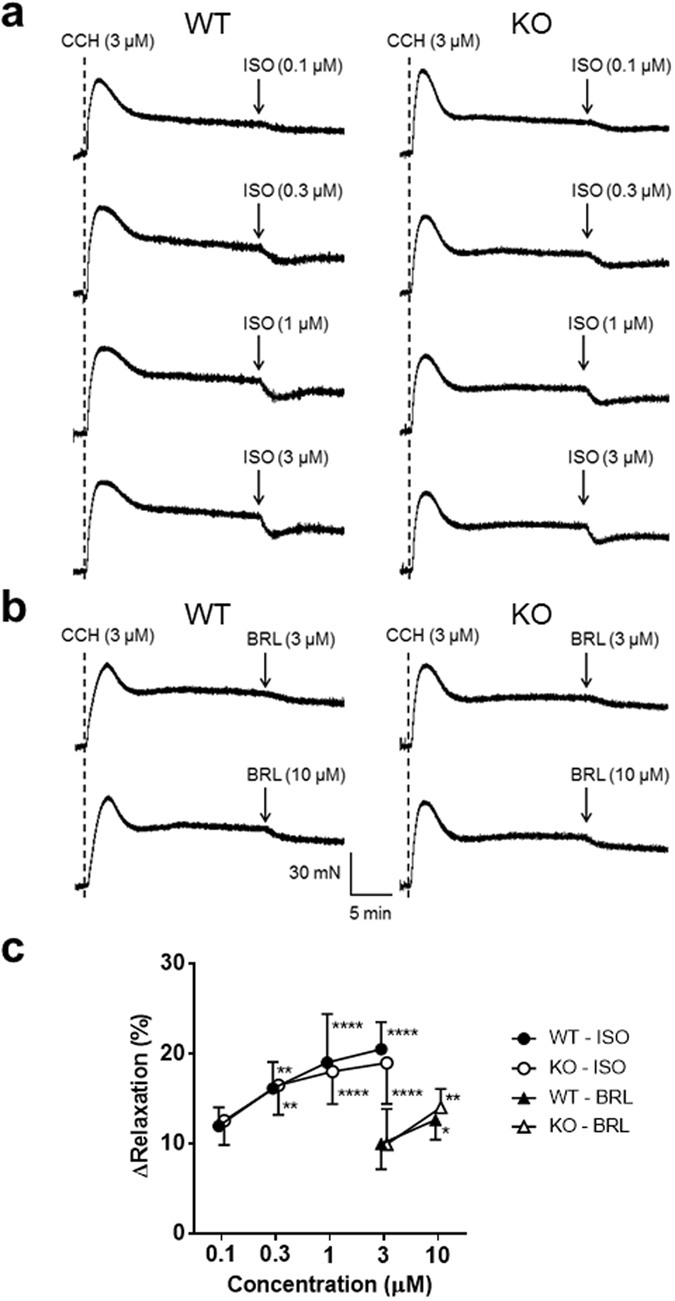
Relaxing effect of a β-adrenergic agonist on pre-contracted *in vitro* bladder muscle strips. (**a**) Effects of graded concentrations of isoproterenol (ISO, 0.1 µM, 0.3 µM, 1 µM, and 3 µM) on carbachol (CCH, 3 µM)-induced pre-contraction of a bladder muscle strip were compared between a WT mouse (left) and a P2Y_6_-KO mouse (right). CCH was given at the timing of the dotted line. (**b**) Likewise, the effect of BRL 37344 (BRL, 3 µM and 10 µM) was compared between a WT mouse (left) and a P2Y_6_-KO mouse (right). There were no differences between the 2 groups in degree of relaxation induced by either isoproterenol (WT, n = 10 and P2Y_6_-KO, n = 12; P = 0.47, repeated measures two-way ANOVA) or BRL 37344 (WT, n = 12 and P2Y_6_-KO, n = 10; P = 0.38) (**c**). Significant difference from 0.1 µM of isoproterenol or 3 µM of BRL 37344: *P < 0.05, **P < 0.01, ****P < 0.0001 (Tukey’s multiple comparisons test for isoproterenol, or Sidak’s multiple comparisons test for BRL 37344). Values are expressed as the mean ± SEM.

Sympathetic innervation to the bladder smooth muscle facilitates storage accommodation during bladder-filling *via* β-adrenoceptors. To examine if P2Y_6_ interacts with β-adrenergic modulation, we compared WT and P2Y_6_-KO mice with respect to the relaxation of pre-contracted bladder strips in response to isoproterenol (non-selective β-agonist) or BRL 37344 (selective β_3_-agonist). Isoproterenol (0.1 µM, 0.3 µM, 1 µM and 3 µM) relaxed 3 µM carbachol-induced contractions of bladder muscle strips of WT and P2Y_6_-KO mice in a concentration-dependent manner (Fig. 5a). Likewise, BRL 37344 (3 µM and 10 µM) produced concentration-dependent relaxation of bladder muscle strips in both groups (Fig. 5b). There was no difference between the 2 groups with respect to bladder muscle relaxation induced by either isoproterenol (P = 0.47, by repeated measures two-way ANOVA) or BRL 37344 (P = 0.38; Fig. 5c).

### Stretch-induced ATP release from primary-cultured urothelial cells

**Figure 6:**
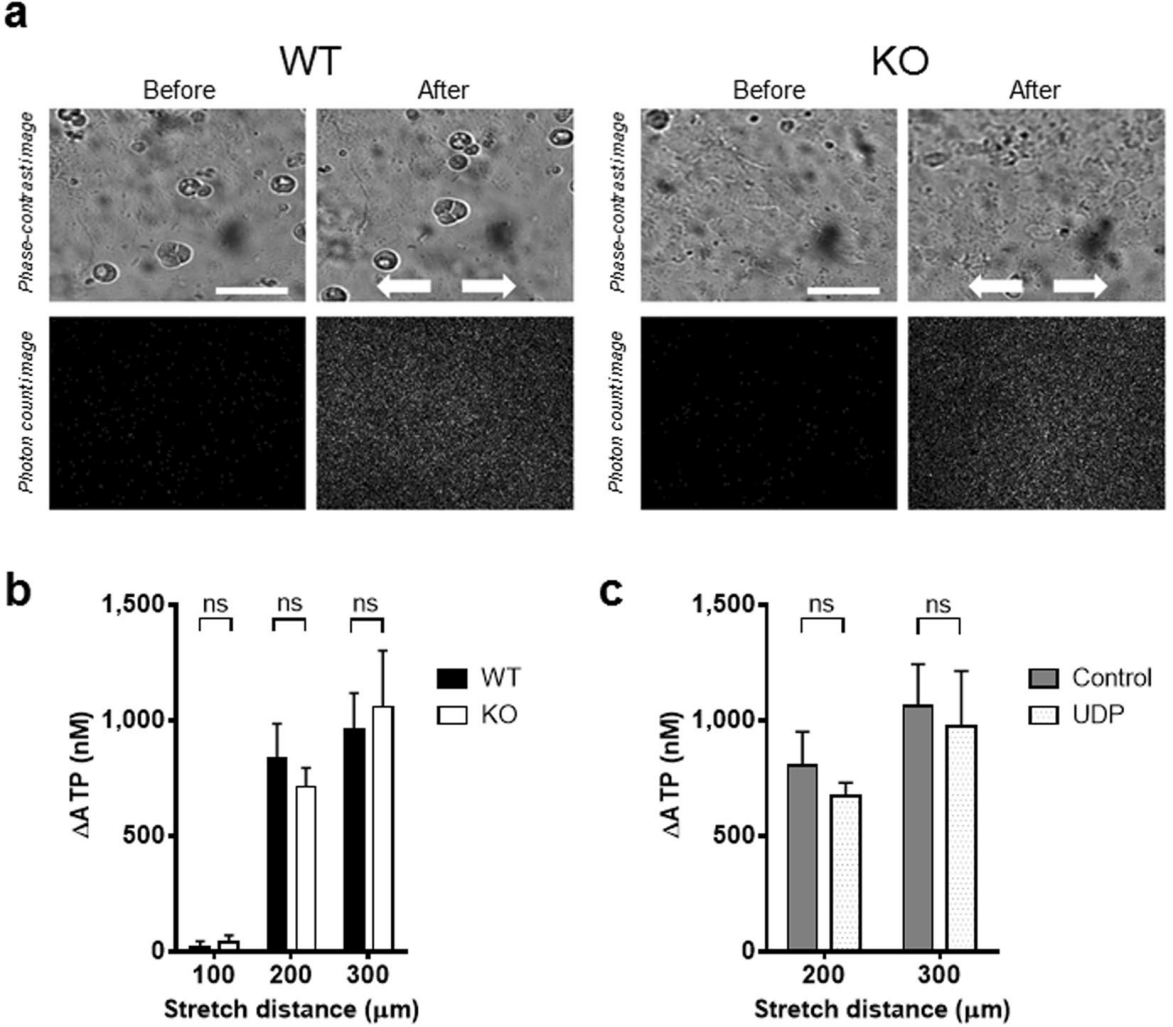
Visualization of stretch-evoked ATP release from primary-cultured urothelial cells. (**a**) The upper panels show urothelial cells in phase-contrast images, and the lower panels show photon count images (white dots) in the same field. The left and right panels show the images before and after mechanical stretch, respectively. The stretch speed was 100 µm/s, and the distance was 200 µm. Cells were extended transversely (indicated by arrows). Scale bar: 100 µm. (**b**) Comparison of stretch-induced ATP release from mouse primary-cultured urothelial cells at different stretch distances (100 µm, 200 µm, and 300 µm) between WT and P2Y_6_-KO cells, and no differences were found between the 2 groups (P = 0.59 at 100 µm, P = 0.91 at 200 µm, P = 0.95 at 300 µm by a Mann-Whitney test). Numbers of data collected for 100 µm, 200 µm, and 300 µm were: 10, 20, and 10 in WT cells, and 9, 19, and 10 in P2Y_6_-KO cells, respectively. (**c**) Comparison of stretch-induced ATP release from WT mouse primary-cultured urothelial cells at different stretch distances (200 µm, and 300 µm) between the absence and presence of UDP (100 µM), and no differences were found between the 2 groups (P = 0.39 at 200 µm, P = 0.77 at 300 µm by an unpaired t-test). Numbers of data collected for 200 µm and 300 µm were: 11 and 16 in control group, and 12 and 15 in UDP group, respectively. ns, not significant.

Bladder urothelium releases ATP upon stretch stimulation, which mediates signalling to primary afferent neurons *via* purinergic receptors^23^. To determine whether activation of P2Y_6_ is required for the stretch-induced ATP release from urothelial cells, we measured the amount of extracellular ATP following mechanical cell stretch stimulation by using an ATP photon imaging system. Urothelial cells on a stretch chamber were extended using the following conditions: stretch distance of 100 µm, 200 µm or 300 µm, and stretch speed of 100 µm/s. Upon stretch stimulation, prominent ATP release occurred in WT cells and P2Y_6_-KO cells, both of which were comparable in amount (Fig. 6a,b). In addition, we compared the stretch-induced ATP release from WT cells between the presence and absence of a P2Y_6_ agonist, UDP (100 µM). As shown in Fig. 6c, the agonist did not affect the ATP release.

## Discussion

Compared with WT mice, P2Y_6_-KO mice showed markedly higher micturition frequency and smaller urination volume/voiding during voluntary voiding behaviour. In reflex micturition cycles during cystometry under decerebrate, unanaesthetized conditions, P2Y_6_-KO mice had markedly smaller bladder capacity than WT mice. No differences in the bladder pressure-volume relationship (i.e., during bladder-filling) and the peak contraction pressures (i.e., during bladder contraction presenting micturition) were found between the 2 groups, whereas the bladder contraction duration was prominently reduced in P2Y_6_-KO mice. There were no differences between the 2 groups with respect to contraction of *in vitro* bladder muscle strips induced by KCl, carbachol, ATP or EFS and with respect to relaxation by a β-adrenergic agonist of pre-contracted bladder muscle strips. Also, no difference in ATP release evoked by stretch stimulation of primary-cultured urothelial cells was found between these groups. Thus, the *in vivo* dual voiding function analysis of voiding behaviour and reflex micturition demonstrated that the P2Y_6_-deficiency is associated with frequent micturition, decreased voiding volume and early attenuation of bladder contractility, whereas the *in vitro* experiments show no apparent involvement of the P2Y_6_ receptor either in contraction and relaxation of bladder smooth muscle or in ATP release from urothelial cells in response to mechanical stimulation.

In response to mechanical stimulation (i.e., bladder distension), urothelial cells in bladder urothelium release ATP that activates P2X_3_ receptors in suburothelial nerve plexus to induce firing of the bladder afferent that conveys the mechanosensory signals to the CNS^24–26^. As an intravesical volume increases, the afferent firing in the pelvic nerve from the bladder gradually increases, which activates spinobulbospinal reflex pathways that pass through the pontine micturition centre (PMC) that functions as an “on-off” switch to trigger micturition^27, 28^. Thus, first of all, it should be considered that the cause of increased micturition frequency and decreased bladder capacity are attributable to increased excitability of afferent sensory pathway or decreased threshold in activation of the PMC in the micturition reflex pathway. Urinary continence is maintained by the sympathetic outflow in the hypogastric nerve from the thoracolumbar sympathetic nucleus to the bladder neck/proximal urethra and by the pudendal outflow from the lumbosacral motor nucleus to the external urethral sphincter^27, 28^. Disruption of the continence mechanism causes early response in releasing the intravesical fluid. Therefore, secondly, possible involvement of urethra function with respect to the frequent micturition and small voiding volume should be also considered.

Our real-time RT-PCR analysis determined gene expression of the P2Y_6_ subtype in urothelium, suburothelium and detrusor (Fig. 1). A previous immunolabelling study demonstrated that, of P2Y receptor subtypes, the P2Y_6_ receptor is most abundantly expressed in urothelial cells of the guinea-pig bladder^29^. These results suggest the possibility that the receptor plays a role in homeostatic or pathophysiological function in the bladder. The present experiments using a previously established ATP measurement technique^30–32^, however, showed that neither P2Y_6_-deficiency nor the P2Y_6_ agonist affects ATP release by stretch stimulation of primary-cultured urothelial cells, suggesting that the urothelial P2Y_6_ receptor is not physiologically involved in mediation of ATP release in response to mechanical stimulation. Thus, deletion of the P2Y_6_ receptor in the urothelium is not likely to be the cause of bladder overactivity in the *in vivo* experiments.

The bladder pressure-volume relationship during cystometry indicated that the P2Y_6_ receptor is not involved in modulation of bladder tone during bladder-filling. *In vitro* experiments examining relaxation of pre-contracted bladder muscle strips in response to the β-adrenergic agonist showed that deletion of the P2Y_6_ receptor does not affect the postsynaptic, sympathetic regulation of bladder muscle tone. These results suggest that the P2Y_6_ receptor is not associated with change in bladder muscle tension that might influence on the bladder afferent firing.

The bladder efferent limb (i.e., pathway that conveys signals from the PMC) that mediates bladder motility is not considered to directly participate in regulation of micturition frequency. ATP and acetylcholine are the major transmitters from the bladder efferent nerve terminals to induce detrusor contraction^25^. It is unknown whether ATP or acetylcholine from the urothelium can directly stimulate the detrusor to contract. Even if there is such mechanism, the present results showed that P2Y_6_-deficiency also does not change the contraction of *in vitro* bladder muscle strips in response to activation *via* ATP or acetylcholine.

The participation of suburothelial myofibroblasts *via* the P2Y_6_ receptor in facilitating propagation of sensory signalling by enhancing individual cellular responses has been proposed^29, 33^. However, it is not likely that this mechanism is involved in the bladder overactivity because deletion of the suburothelial P2Y_6_ receptor would be expected to reduce the bladder afferent firing.

*In vivo* micturition variables are greatly influenced, not only by bladder activity, but also by urethra function^34–36^. In the present study, voluntary voiding behaviour analysis showed that there is no difference between WT and P2Y_6_-KO mice in uroflow rate (Fig. 2h) and cystometry evaluation revealed that post-void residual volume is not increased in P2Y_6_-KO mice, compared with WT mice (Table 1). These results showed that the coordinated expelling function of the bladder and urethra is well preserved in P2Y_6_-KO mice, thus suggesting that systemic deletion of the P2Y_6_ receptor does not disturb micturition.

The previous study by Otomo and co-workers using dual recording of isovolumetric bladder pressure and simultaneous urethral pressure in urethane-anaesthetized rats showed an intra-arterial (i.a.) injection of RB-2, a non-selective P2Y antagonist, increases urethral resting pressure (i.e., urethral tone relative to bladder-filling phase during cystometry) but does not change maximal urethral relaxation (i.e., decreased urethra pressure relative to the bladder contraction/voiding phase during cystometry)^37^, suggesting the possibility that blockade of P2Y receptors in urethra facilitates urinary continence and does not disturb micturition. The latter (i.e., no disturbance in micturition) supports our present result and the former (i.e., facilitation in continence) suggests that blockade of the P2Y_6_ receptor in urethra would not be involved in frequent micturition, although not only the P2Y_6_ but also other P2Y receptor subtypes are non-selectively blocked in their study.

Taken together, our results strongly suggest that the site responsible for the bladder activity caused by systemic deletion of the P2Y_6_ receptor is present, not in bladder and urethra, but in the CNS, dorsal root ganglion (DRG) or both that is involved in the micturition reflex pathway.

Previous studies from other laboratory showed that an intravesical infusion of PSB0474, a P2Y_6_ agonist, increases micturition frequency during cystometry in urethane-anaesthetized rats^16, 17^. The proposed mechanism for the bladder overactivity was that ATP release from the urothelium facilitated by the agonist activates P2X_3_ receptors in suburothelial afferent nerves and causes the hyperreflexia. Controversy may be raised with respect to the inconsistency between these studies with the agonist and our present results using P2Y_6_-KO mice, both of which show bladder overactivity. However, the discrepancy can be attributable to the species difference because the results of our experiments measuring stretch-induced ATP release from primary-cultured urothelial cells revealed that the P2Y_6_ receptor in the mouse urothelium is not important in mediation of the ATP release. Also, in contrast with their studies showing that the prominent bladder overactivity is induced by activation of the P2Y_6_ receptor only in bladder and urethra, our results demonstrating the hyperreflexia associated with systemic deletion of the P2Y_6_ receptor suggest the importance of the receptor involvement in the CNS or the PNS.

Moreover, bladder overactivity or instability generated by the locally-administered P2Y_6_ agonist may be owing to decrease in urethral tonus^38–40^, in addition to activation by the increased intravesical ATP of the suburothelial P2X_3_-signalling. ATP induces relaxation of urethra smooth muscle in various animal species^41–45^. The previous study by Otomo and co-workers demonstrated that i.a. injection of ATP decreases urethral resting pressure (i.e., urethra pressure to maintain continence) without affecting urethral relaxation during a bladder contraction^37^. Activation of urethral afferent pathway by urine leakage into the proximal urethra/bladder neck facilitates a voiding reflex^46^, thus suggesting that a decrease in urethral tonus during bladder-filling would result in early release of the bladder fluid. In agreement with this urethral mechanism, i.a. administration of sodium nitroprusside, a nitric oxide donor, which induces urethral relaxation with neither affecting bladder contractility nor stimulating bladder afferent transmission, produced prominent bladder overactivity in conscious rats^34^. Similarly, α-bungarotoxin, which produces urethral striated muscle relaxation without affecting autonomic bladder pathways, given i.v. markedly increased micturition frequency in decerebrate, unanaesthetized rats^35^. Thus, it is suggested that intravesical ATP release facilitated by the P2Y_6_ agonist, which is perfused into intra-urethra during each voiding, induces urethral relaxation, resulting in bladder overactivity.

The P2Y_6_ receptor is likely to be involved in modulation of bladder contraction force and subsequent relaxation. The detailed analysis of bladder contraction during cystometry revealed that P2Y_6_-KO mice had shorter durations at phases of bladder pressure increase that leads to micturition (i.e., p1 of BCD), bladder pressure decrease accompanying fluid release (i.e., p2 of BCD), and bladder pressure decrease that terminates the contraction (i.e., p4 of BCD) compared with WT mice, whereas these mice showed no difference from WT mice at the phase of bladder pressure increase immediately after expelling the fluid (i.e., p3 of BCD). Shorter duration in p1 or p4 is due to direct or indirect influence of P2Y_6_-deficiency on bladder smooth muscle, whereas that in p2 is likely to be largely caused by smaller voiding volume in P2Y_6_-KO mice.

The shorter p4 duration in P2Y_6_-KO mice is supported by the previous study by Yu and co-workers^18^. The investigators showed that activation of the P2Y_6_ receptor enhances the P2X_1_-mediated contractile force and induces a sustained increase in *in vitro* bladder smooth muscle tone, further suggesting that the synergistic interaction between the P2X_1_ response and P2Y_6_ signalling is conducted through the phospholipase C (PLC)/inositol trisphosphate (IP3) pathway. In agreement with their result^18^, the present cystometry experiments showed that bladders of P2Y_6_-KO mice are unable to sustain the contraction force for as long a duration as those of WT mice. In addition, it is possible that the bladder relaxation during p4 of P2Y_6_-KO mice is indirectly enhanced by altered contributions of other P2Y subtypes due to the P2Y_6_-deficiency. For example, P2Y_1_ mRNA is expressed in rat urinary bladders^47^ and an activation of P2Y_1_ inhibits acetylcholine release from parasympathetic, cholinergic nerve terminals^48^, which would change bladder contractility. P2Y_1_ receptor mRNA is markedly increased in the aorta of P2Y_6_-KO mice^49^. Likewise, P2Y_1_ expression may be increased in urinary bladders of P2Y_6_-KO mice, resulting in augmented implications of the receptor. Upon the use of genetically mutated animals, it is important to consider the possibility that other genes that have not been deliberately manipulated can be subsequently affected.

The results of the present study suggest that the P2Y_6_ phenotypes of the mouse lower urinary tract function are associated with an inhibition of excitatory bladder afferent signalling or of sensitivity in the PMC and with a facilitation of sustaining bladder contraction force. The P2Y_6_ receptor in the CNS (not including the forebrain), DRG or both is implicated in regulation of urinary frequency, whereas that in the detrusor is likely to be involved in modulation of bladder contractility. The P2Y_6_ receptor has been found in the DRG^12^ and the spinal dorsal horn^10^, indicating that the receptor is involved in sensory transmission. In fact, previous studies showed central and peripheral involvements of the P2Y_6_ receptor in development of neuropathic pain^10, 12^; however, it is unknown whether the receptor participates in normal sensory transmission. The use of P2Y_6_ receptor antagonists may be promising in alleviating neuropathic pain and symptoms associated with inflammation and neurodegeneration^10, 12–15^; while, our study claims that great attention must be paid to development of the pharmacotherapy for possible adverse impact of frequent urination. Further study is necessary to determine the P2Y_6_ site responsible for controlling the lower urinary tract function. Moreover, it is of interest to investigate, in other species including humans, whether malfunction or blockade of the P2Y_6_ receptor generates lower urinary tract symptom such as bladder overactivity.

## Materials and Methods

### Animals

All animals in this study were obtained, housed, cared for and used in accordance with the “Guiding Principles in the Care and Use of Animals in the Field of Physiologic Sciences” published by the Physiologic Society of Japan. In addition, all experimental protocols were approved by the Institutional Animal Care and Use Committee of the University of Yamanashi (Chuo, Yamanashi, Japan). All efforts were made to minimize animal suffering and reduce the number of animals used.

Experiments were conducted using 8- to 12-week-old male C57BL/6N mice (SLC, Shizuoka, Japan). A pair of P2Y_6_-KO mice were generously provided by Dr. Bernard Robaye (Universite Libre de Bruxelles, Brussels, Belgium). These mice were backcrossed (for more than 8 generations) on a C57BL/6N background. Before each experiment, the mice were housed under a 12:12-h light-dark cycle with controlled humidity and temperature, and they were provided with food and water *ad libitum*.

### Quantitative real-time PCR

Quantitative real-time PCR assays were performed on mouse specimens as previously described^50^. The primer sequences for amplification are shown in Supplementary Table 1S.

### Analysis of voluntary voiding pattern in metabolic cages

Evaluations of voluntary voiding behaviour were conducted according to a previously published method^20^. In brief, conscious mice were individually placed in novel, patented metabolic cages (Shinfactory Co. Ltd., Fukuoka, Japan) constructed in a soundproof room at 25 °C with a 12:12-h light-dark cycle. Each mouse was provided with free access to food and water. After an acclimation period of 3 days in the cage, data on voided urine (weight and timing) and water consumption (volume and timing) were continuously collected for each mouse over 2 days using a Power-Lab data-acquisition system (AD Instruments, Colorado Springs, CO, USA).

### Evaluation of reflex micturition cycle during cystometry

Urodynamic analysis was conducted in decerebrate, unanaesthetized mice according to a previously published method^20^. Animals were anaesthetized with sevoflurane (2.5–5%) in O_2_ (flow rate: 0.2 l/min) during surgery. After a tracheal cannulation with a polyethylene tube (PE-90, Clay-Adams, Parsippany, NJ) to facilitate respiration, the precollicular decerebration was performed^51^: both carotid arteries are ligated, followed by a midline incision of the head skin with a scalpel and by removal of the skull and the forebrain with a fine rongeur and a blunt spatula, respectively. Sevoflurane was then discontinued. After no further intracranial hemorrhage was detected visually, the lateral flaps of the incised head skin were sutured together. Cystometry was performed by a continuous infusion of room temperature physiological saline (10 µl/min) into the bladder *via* the dome to elicit repetitive voids, which allowed for data collection from a number of micturition cycles. Fluid voided from the urethral meatus was collected and measured to determine voided volume. Once constant voided volumes were collected, the infusion was stopped at the beginning of a voiding contraction, and the last voided volume was measured^35^. Immediately after the final voided volume measurement, the abdomen was opened by suture removal, and the bladder was exposed. The remaining intravesical content was expelled by directly exerting pressure on the bladder with a curved forceps, and the collected fluid was estimated as residual volume.

### Histopathological examination

Whole bladders excised from mice were embedded in tissue-TEK OCT compound (Sakura Finetek, Tokyo, Japan), frozen in liquid nitrogen, and cut into 7 µm sections. The bladder tissues mounted on glass slides were fixed with 4% paraformaldehyde, and then stained with haematoxylin and eosin (H&E) for microscopic examination.

### Isometric contraction or relaxation of *in vitro* bladder muscle strips

Body of bladder was excised and then bladder strips approximately 5 to 6 mm wide and 7 to 10 mm long were prepared. The bladder strips with intact mucosa were suspended in a 15-ml organ bath (Panlab, Barcelona, Spain) filled with Krebs solution composed of 119 mM NaCl, 4.6 mM KCl, 1.5 mM CaCl_2_, 1.2 mM MgCl_2_, 15 mM NaHCO_3_, 1.2 mM NaH_2_PO_4_, and 5 mM glucose at 37 °C and gassed with 95% O_2_/5% CO_2_. Changes in strip tension were measured with an MLT0210/D isometric force transducer (AD Instruments) and recorded with an ML118 and ML785 Power-Lab acquisition system (AD Instruments). Strips were placed between two platinum electrodes in the organ baths containing Krebs solution and an initial tension of 1 g was applied to the strips, which were allowed to equilibrate for at least 30 min before experiments. Strips were first challenged with 60 mM KCl to test tissue viability.

In contraction experiments, the strips were stimulated by carbachol, electrical field stimulation (EFS) at frequencies of 1 Hz, 2 Hz, 4 Hz, 8 Hz, 16 Hz, and 32 Hz with and without 10 µM atropine, and 1 mM ATP, in this order. Prior to each stimulation, washout with Krebs solution was performed 3 times. Carbachol concentration-response curves were constructed by adding graded concentrations, which were expressed as the final concentration of 3 nM to 100 mM in the organ bath. Contractile responses in strips were normalized to the weight of each strip.

In relaxation experiments, prior to testing each drug concentration, the strips were stimulated by 3 µM carbachol for evoking its contraction. When contraction became stable, a non-selective β-adrenoceptor agonist, isoproterenol (0.1 µM, 0.3 µM, 1 µM and 3 µM) or a selective β_3_-adrenoceptor agonist, BRL 37344 (3 µM and 10 µM) was applied. Each strip was treated with either drug. Prior to each drug concentration, washout with Krebs solution was performed 3 times. Relaxing responses in strips were normalized to the weight of each strip. Maximal relaxation of the tension was used for data analysis, which were expressed as a percent inhibition of the carbachol-induced tension of the bladder strip.

### Measurement of ATP release from primary-cultured urothelial cells

Preparation of urothelial cultures, mechanical stretching experiments and photon imaging of ATP release were performed as previously described^30, 31^.

In brief, an elastic silicone stretch chamber (STB-CH-04, STREX, Osaka, Japan) and an extension device (STB150, STREX) were set on a photon imaging system, and the chamber medium was replaced with extracellular solution containing a luciferase reagent (ATP bioluminescence assay kit CLS II, Roche Diagnostics, Basel, Switzerland). ATP bioluminescence during stretch stimulation was detected and visualized with a VIM camera (C2400-30H, Hamamatsu Photonics, Hamamatsu, Japan). The standard calibration curve yielded a correlation coefficient for bioluminescence *vs.* ATP concentration of 0.998 over a concentration range of 0 nM to 2 μM.

### Drugs

ATP (Sigma-Aldrich Japan GK, Tokyo, Japan), atropine (Sigma-Aldrich Japan GK), carbachol (Sigma-Aldrich Japan GK), KCl (Nacalai Tesque, Inc., Kyoto, Japan), isoproterenol (Sigma-Aldrich Japan GK) and BRL 37344 (Sigma-Aldrich Japan GK) were used for *in vitro* experiments using bladder muscle strips. UDP (Sigma-Aldrich Japan GK) was used for experiments measuring ATP release from primary-cultured urothelial cells. Sevoflurane (Maruishi Pharmaceutical, Osaka, Japan) was used to anaesthetize animals during surgery for cystometry experiments.

### Statistical analysis

All values are expressed as the mean ± SEM. The Wilcoxon matched-pairs signed rank test, Mann-Whitney test, unpaired t-test, F-test and repeated measures two-way analysis of variance (ANOVA) followed by Tukey’s multiple comparisons test or Sidak’s multiple comparisons test were used for statistical analysis, if applicable. Coefficients (r) and P values (P) were calculated by Spearman’s correlation analysis or by Pearson’s correlation analysis, if applicable, to examine correlations between variables. For all analyses, P values of <0.05 were considered significant.

## Electronic supplementary material

Supplementary
