# Supplementary material for: P2Y6-deficiency increases micturition frequency and attenuates sustained contractility of the urinary bladder in mice
Source: Sci Rep. 2017 Apr 10;7:771. doi: 10.1038/s41598-017-00824-2 (PMC5429706; doi:10.1038/s41598-017-00824-2)
Supplement: Supplementary file 1 — Supplementary [file 41598_2017_824_MOESM1_ESM.pdf]

Supplementary Information

P2Y$_6$-deficiency increases micturition frequency and attenuates sustained contractility of the urinary bladder in mice

Satoru Kira[1], Mitsuharu Yoshiyama[1], Sachiko Tsuchiya[1], Eiji Shigetomi[2,3], Tatsuya Miyamoto[1], Hiroshi Nakagomi[1], Keisuke Shibata[2,3], Tsutomu Mochizuki[1], Masayuki Takeda[1] & Schuichi Koizumi[2,3]

[1]Department of Urology, University of Yamanashi Graduate School of Medical Science, Yamanashi 409-3898, Japan
[2]Department of Neuropharmacology, University of Yamanashi Graduate School of Medical Science, Yamanashi 409-3898, Japan
[3]Japan Science and Technology Agency, CREST, Tokyo 102-0076, Japan

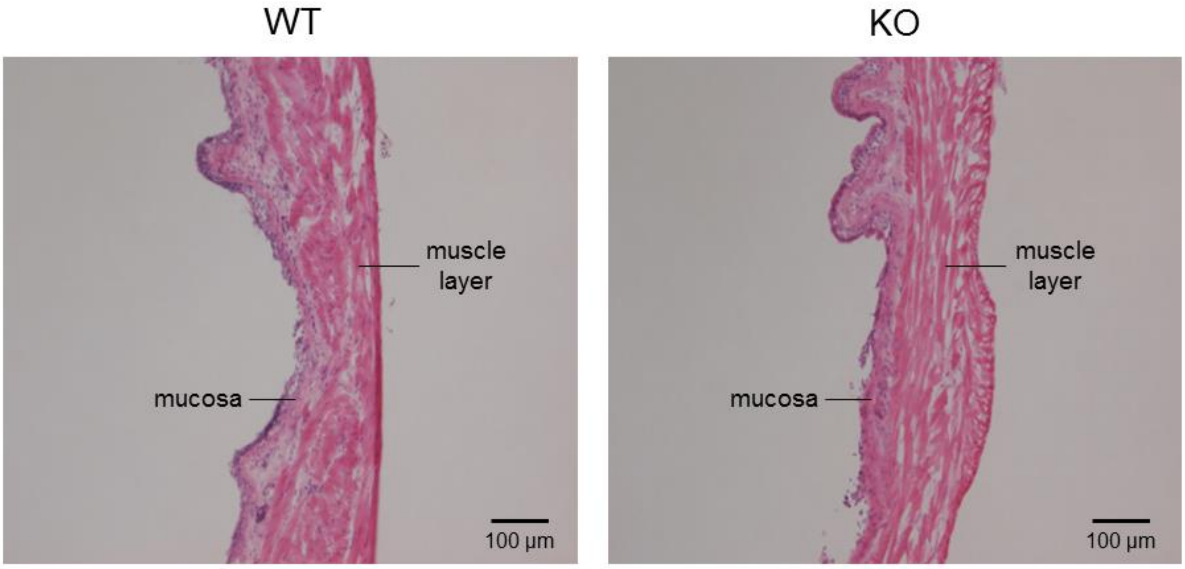

Figure 1S. Microscopic findings of bladders stained with haematoxylin and eosin (H&E), of a WT mouse and a $P2Y_6$-KO mouse

Table 1S. The primer sequences for RT-PCR

Probe	Sequence
$P2Y_1$	
forward	CTGGGACTCGGAAAAACAAA
reverse	AGATCAGCACCAAAGGGATG
$P2Y_2$	
forward	CTGTCACCACCCACACAAAG
reverse	ACACACACACACACCCATCC
$P2Y_4$	
forward	TCCTTTTCCTCACCTGCATC
reverse	AGGCAGCCAGCTACTACCAA
$P2Y_6$	
Forward	AGGCTGACAGGCAGTTATGG
Reverse	TATACCGGGGTTAGCAGCAG
$P2Y_{12}$	
forward	GGCTTTGGGAACTTATGCAA
reverse	GTGTTGACACCAGGCACATC
$P2Y_{14}$	
forward	GAAGAATTCCACCTCCGTCA
reverse	GCACAAAGCAGACGACAAAA
$P2X_1$	
forward	TGTACGGGGAGAAGAACCTG
reverse	TCCCAAACACCTTGAAGAGG
GAPDH	
forward	CACAATTTCCATCCCAGACC
reverse	GTGGGTGCAGCGAACTTTAT
